# Cheese as Food

**Published:** 1875-04

**Authors:** 


Cheese as Food.—It is evident that cheese might be made more available for food than it is at present. The analysis of cheese shows that it contains about twice as much flesh-forming or life-sustaining substance as butcher's meat, but it does not practically take the place of meat to that extent. There are objections to the use of cheese because of improper care of the milk of which it is made, and of faulty modes in manufacturing and curing. Bad qualities are allowed to get into the milk by way of bad food and water, and errors in the treatment of milk and curds develop an excess of fermentation, curing the cheese too fast and changing the character of the fermentation, often making it unwholesome. The fermentation which goes on in a cheese in its best estate makes it impossible for some people to use it. It becomes an actual poison. These cases are rare. But when the fermentation becomes modified and excessive by reason of the introduction or development of unusual agents, the number to whom cheese becomes distasteful and objectionable is very greatly increased. On the other hand, large quantities of cheese are now offered as food which are objectionable from a lack of fermentation.

Curd, either in its green or dried state, is not easily digested in large quantities, unless minutely divided and commingled with other food. It is more soluble in alkali than in acid, and therefore the acids of the gastric juice act upon it but feebly. If curd or cheese is thoroughly masticated, in connection with some hard food, the alkali of the saliva will greatly assist the digestive process. Cheese may be eaten in this manner with comfort by nearly all persons. Cheese should be very slowly cured, in order that the peculiar fermentive process which renders it so valuable as an article of diet may be perfectly accomplished.

---

Why should people read? and what is the real, solid value, of printed matter? There are three good reasons for reading, and we can think of no others. They are: to be made wiser, to be made nobler, and to be innocently recreated. Books which neither confer information which is worth having, nor lift the spiritual part of us up to loftier regions, nor, by judicious diversion, refreshen the mind for further serious efforts, are bad books, and the reading of such is invariably idleness, and usually the most dangerous kind of idleness. Reading is not, as so many people nowadays seem to suppose, good in itself, as so many things are which are by no means as highly thought of. All energy that is not injurious, wasteful, or subtracted from some other effort incumbent upon him who puts it forth, is good,—as walking, riding, boating, and the rest. But the reading of which we speak can not, under the most favorable construction, be regarded as energy. On the contrary, it is the very laziest form of laziness. People fly to it when they think they have nothing else to do, and they flatter themselves that by reading they are really doing something; and thus, nine times out of ten, they exonerate themselves from the obligation of performing some genuine duty which is distasteful to them.

